# A Cultivar-Sensitive Approach for the Continuous Monitoring of Olive (*Olea europaea* L.) Tree Water Status by Fruit and Leaf Sensing

**DOI:** 10.3389/fpls.2020.00340

**Published:** 2020-03-24

**Authors:** Alessio Scalisi, Giulia Marino, Francesco Paolo Marra, Tiziano Caruso, Riccardo Lo Bianco

**Affiliations:** ^1^Department of Agricultural, Food and Forest Sciences (SAAF), University of Palermo, Palermo, Italy; ^2^Agriculture Victoria, Department of Jobs, Precincts and Regions, Tatura, VIC, Australia; ^3^Department of Plant Sciences, University of California, Davis, Davis, CA, United States

**Keywords:** drought, fruit diameter, sustainable irrigation, turgor pressure, water deficit, water potential

## Abstract

Sustainable irrigation is crucial to reduce water use and management costs in modern orchard systems. Continuous plant-based sensing is an innovative approach for the continuous monitoring of plant water status. Olive (*Olea europaea* L.) genotypes can respond to drought using different leaf and fruit physiological and morphological mechanisms. This study aimed to identify whether fruit and leaf water dynamics of two different olive cultivars were differently affected by water deficit and their response to changes of midday stem water potential (Ψ_stem_), the most common indicator of plant water status. Plant water status indicators such as leaf stomatal conductance (g_s_) and Ψ_stem_ were measured in the Sicilian olive cultivars Nocellara del Belice (NB) and Olivo di Mandanici (MN), in stage II and III of fruit development. Fruit gauges and leaf patch clamp pressure probes were mounted on trees and their raw data were converted in relative rates of fruit diameter change (RR_fruit_) and leaf pressure change (RR_leaf_), sensitive indicators of tissue water exchanges. The analysis of diel, diurnal and nocturnal fluctuations of RR_fruit_ and RR_leaf_ highlighted differences, often opposite, between the two cultivars under water deficit. A combination of statistical parameters extrapolated from RR_fruit_ and RR_leaf_ diurnal and nocturnal curves were successfully used to obtain significant multiple linear models for the estimation of midday Ψ_stem_. Fruit and leaf water exchanges suggest that olive cultivar can either privilege fruit or leaf water status, with MN likely preserving leaf water status and NB increasing fruit tissue elasticity under severe water deficit. The results highlight the advantages of the integration of fruit and leaf water dynamics to estimate plant water status and the need for genotype-specific models in olive.

## Introduction

In recent years, sustainable irrigation has become a crucial aspect of orchard management to reduce inputs in agricultural systems. In the current global warming and desertification scenario, both environmentally and economically oriented reasons provide the basis for a water saving approach, which has become paramount in irrigated orchards. Automated irrigation management is even more important in high-density systems in which growers tend to increase orchard productivity and reduce management costs by mechanizing operations. In the past, irrigation management was commonly based on soil water status or environmental indices. However, tree water status provides the most precise drought stress indices, in spite of soil and environmental conditions. Indeed, plants represent the intermediate component of the soil-plant-atmosphere continuum, and their physiological responses are the result of an integration of both soil and environment. This implies an advantage of plant-based over soil-based methods for an accurate irrigation scheduling (Fernández, 2017).

Plant water requirements differ among species and even cultivars, making irrigation scheduling and management a complex task for growers. The physiological responses of plants to decreasing water availability are various and depend on evolutionary adaptation and acclimation to new climatic conditions. The olive species (*Olea europaea* L.) has a very wide genetic pool, and includes genotypes that can respond to drought using different mechanisms of leaf dehydration tolerance and leaf morphological and structural adaptations (Bacelar et al., 2004, 2006; Ennajeh et al., 2010; Lo Bianco and Scalisi, 2017; Scalisi et al., 2019a). Gucci et al. (2000) and Lo Bianco and Scalisi (2017) found different leaf stomatal regulation among olive cultivars. Connor (2005) suggested that olive genotypes use avoidance (i.e., low leaf conductance, low leaf area, low xylem hydraulic conductivity and large roots) and tolerance mechanisms (i.e., high turgor through cell osmotic adjustments, small cell size, and changes in cell-wall elasticity) under drought. Midday stem water potential (Ψ_stem_) is considered a very sensitive parameter of plant water status for irrigation management (Moriana and Fereres, 2002; Fernández et al., 2006; Moriana et al., 2012, Marino et al., 2018). However, Ψ_stem_ is mostly measured by the Scholander pressure chamber, which does not allow for continuous monitoring and automated irrigation.

Recently, plant-based sensing technologies are taking hold for the continuous plant water status monitoring in fruit trees. In most of cases, sensors are mounted on aboveground organs such as stem, fruit and leaves (Fernández, 2014, 2017; Scalisi et al., 2017). In olive, trunk dendrometers have been associated with tree water stress thresholds and proposed for irrigation management due to their relatively easy installation and stability across the season (Moreno et al., 2006; Cuevas et al., 2010; Fernández et al., 2011b).

In the last few years, emphasis has been given to the use of leaf patch clamp pressure (LPCP) probes for the continuous assessment of olive leaf water status (Fernández et al., 2011a; Ehrenberger et al., 2012; Rodriguez-Dominguez et al., 2012, 2019; Marino et al., 2016; Padilla-Díaz et al., 2016; Fernándes et al., 2017). The output of LPCP probes is expressed as attenuated pressure of leaf patches (p_p_), which is inversely related to cell turgor pressure (p_c_) (Zimmermann et al., 2008). Therefore, the highest values of p_p_ occur around solar noon, as that is the moment in which leaf cell turgor is the lowest. Ben-Gal et al. (2010) found an inversion of the p_p_ curve in severely drought-stressed olive trees. Thereafter, Fernández et al. (2011a) classified water deficit states based on the degree of inversion of the curve. State I represented no drought stress and leaves with a non-inverted curve, state II grouped leaves experiencing partial inversion of the curve and mild water deficit, and state III enclosed all leaves experiencing severe water deficit and full inversion of the curve.

Fruit-based probes based on linear variable displacement transducers (LVDTs) can provide good information on fruit growth, which on a diel scale is mostly dominated by water in- and out-flows, rather than carbon gain; thus, fruit diameter (FD) variations respond to water deficit (Scalisi et al., 2017). Fernandes et al. (2018) studied olive FD dynamics in response to water deficit, suggesting the appropriateness of fruit gauges for continuous plant water status monitoring. Although FD and p_p_ are strictly related to soil water availability and plant water status, they are also influenced by environmental variables, crop load, genetic factors and phenology. The derived values of FD and p_p_ (namely relative rate of fruit diameter change, RR_fruit_ and relative rate of leaf pressure change, RR_leaf_) represent good indicators of the rate at which water enters and exits leaf or fruit, respectively. The reasoning behind this assumption resides in the fact that they both represents rates of changes – pressure and diameter, respectively – from an initial state, which, in the short term (≤15-minute intervals) are mainly driven by tissue water exchanges. The combined use of RR_fruit_ and RR_leaf_ was recently introduced to estimate water deficit in nectarine (Scalisi et al., 2019b, c). Plants modulate water movements to and from the two main transpiring organs (i.e., leaf and fruit) using several strategies such as osmotic adjustments, stomatal closure or cell-wall elasticity regulation. As a result, we hypothesized that either leaf or fruit water status might be privileged under increasing water deficit in olive genotypes with different drought tolerance/avoidance mechanisms.

This work aimed to study olive fruit and leaf water dynamics in relation to tree water status. Our hypothesis was that, similarly to what found in nectarine (Scalisi et al., 2019c), the combination of RR_fruit_ and RR_leaf_ might provide an even more accurate identification of plant water status, rather than monitoring each parameter independently. In addition, this study aimed to identify cultivar-specific RR_fruit_/RR_leaf_ relationships to determine whether the genotypes under study preserve leaf or fruit water exchanges under increasing water deficit, as sink power for water might differ among genotypes.

## Materials and Methods

### Experimental Design

The experiment was carried out in summer 2016 in a high-density (6 × 3 spacing, ∼ 555 trees/ha) olive orchard located near Sciacca, in South-western Sicily (37°29′56.8″ N and 13°12′13.4″ E, 138 m a.s.l.). Three-year-old self-rooted trees were trained to “free palmette” along a hedgerow in North-to-South rows. The experimental orchard is part of a large survey on the Sicilian autochthonous germplasm (Marino et al., 2019). Olive alternates high and low cropping seasons and this experiment considered only trees in an ON-year (heavy crop load). In this trial, the two Sicilian cultivars Nocellara del Belice (NB) and Olivo di Mandanici (MN) were selected for their different vigor and fruit characteristics (Marino et al., 2016, 2017). Trees belonging to NB have low vigor, spreading canopy habit and yield large fruit, mainly processed as table olives, whereas MN trees show high vigor, upright canopy habit and yield smaller fruit, exclusively utilized for olive oil extraction. NB trees are particularly sensitive to leaf dehydration (Lo Bianco and Scalisi, 2017) and to water deficit overall (Scalisi et al., 2019a). The soil was a sandy clay loam (60% sand, 18% silt, and 22% clay) with pH of 7.7 and <5% of active carbonates. Trees were regularly fertilized and pruned according to conventional practices.

Meteorological data were retrieved from the meteorological station of Sciacca (Servizio Informativo Agrometeorologico Siciliano). Reference evapotranspiration (ET_0_) and vapor pressure deficit (VPD) were calculated using the methods described by Allen et al. (1998). Crop evapotranspiration (ET_c_) was estimated by weighing ET_0_ with an average K_c_ of 0.50 ± 0.05 (Allen et al., 1998).

Trees were irrigated at weekly intervals using self-compensating in-line drippers delivering 16 L/h. Four irrigation levels were imposed to generate a large variability in tree water status: full irrigation (FI) supplied with a volume of water equal to 100% of ET_c_, and three sustained deficit irrigation levels at 66% (DI-66), 33% (DI-33), and 0% (DI-0) of FI. Six, four, two and no drippers per plant were used for FI, DI-66, DI-33, and DI-0, respectively. Trees were arranged according to a completely randomized experimental design, with twelve tree-replicate per cultivar, and three trees for each irrigation level. Measurements were carried out at stages II (pit-hardening) and III (cell enlargement) of fruit development, as during the stage I (mid-May/beginning July) spring rainfall saturated the soil and canceled any possible effect of deficit irrigation. Data are reported in local time.

### Plant Water Status

Leaf stomatal conductance (g_s_) was measured using a Delta-T AP4 dynamic porometer (Delta-T Devices LTD, Cambridge, United Kingdom) on three sun-exposed leaves in three trees per irrigation level. Daily measurements of g_s_ were taken at 2-hour intervals, from 0800 to 2000 h in a day at stage II (day of the year, DOY = 209) and from 0800 to 1800 h in a day at stage III (DOY = 287).

A pressure chamber (PMS Instrument Co., Corvallis, OR, United States) was used for the determination of Ψ_stem_, on twigs covered by plastic and aluminum foil 1 h before measurement, as described by Turner (1988). Daily measurements of Ψ_stem_ were carried out on three twigs per tree, on the same trees and DOY of g_s_ measurements, at 2-hour intervals and from pre-dawn (0430 h) to 2000 h at DOY 209, and to 1800 h at DOY 287. Midday Ψ_stem_ was measured at around 1400 h, once a week from 196 to 287 DOY, and using three twigs per tree.

### Fruit- and Leaf-Based Sensing

The fruit gauges based on LVDT sensors described by Morandi et al. (2007) were installed on olive drupes for continuous FD measurements. Gauges were wired to four CR-1000 data loggers (Campbell scientific, Inc., Logan, UT, United States) and data were downloaded manually. A total of 16 gauges (eight on each cultivar, two trees per irrigation level) were mounted on sun exposed fruit at medium canopy height (1.5 m above ground). In addition, early in the morning, LPCP probes (Yara International, Oslo, NO, United States) were clamped on sun exposed, mature leaves for continuous measurement of p_p_. LPCP clamping was done a day after irrigation to ensure optimal leaf turgor, carefully avoiding central leaf nerves and placing the piezoresistive sensor on the abaxial side of leaves. The initial LPCP clamping pressure ranged from 15 to 25 kPa. Data of p_p_ were recorded continuously and sent to an online server through a system equipped with radio transmitters and a main GPRS/radio controller. LPCP probes were mounted on leaves nearby the fruit monitored with fruit gauges, using the same number of sensors (i.e., 16). Both fruit gauges and LPCP probes were mounted on the same trees used for g_s_ and Ψ_stem_ measurements.

Fruit gauges and LPCP probes were set to record FD and p_p_ at 15-minute intervals for 8 days at fruit growth stages II and III. A buffer period corresponding to the first 3 days after sensor mounting was discarded to allow adjustments and/or re-clamping in fruit and leaves. Raw FD and p_p_ data were processed using a 15-point convoluted spline function (Savitzky and Golay, 1964) to smooth the sensor signal and erase noise. Following data filtering, FD and p_p_ values were standardized by using *z*-scores [i.e., *z* = (*x*−mean)/standard deviation) to allow comparison among fruits with different initial diameter or leaf with different initial turgor pressure when sensors were mounted. After standardization, FD and p_p_ obtained from different sensors on the same tree were averaged. Second derivatives of FD and p_p_ were calculated to determine RR_fruit_ and RR_leaf_ as shown in Eqs. 1, 2, respectively. A standardization of RR_fruit_ and RR_leaf_ was not carried out as they are based on standardized FD and p_p_, allowing possible comparisons among outputs from different sensors.

(1) RR=fruit(lnFD-2lnFD)1/(t-2t)1

(2) RR=leaf(lnp-p2lnp)p1/(t-2t)1

where, FD_2_ and FD_1_ are FD at time 2 (t_2_) and 1 (t_1_), and p_p__2_ and p_p__1_ are p_p_ at t_2_ and t_1_, respectively.

**FIGURE 1:**
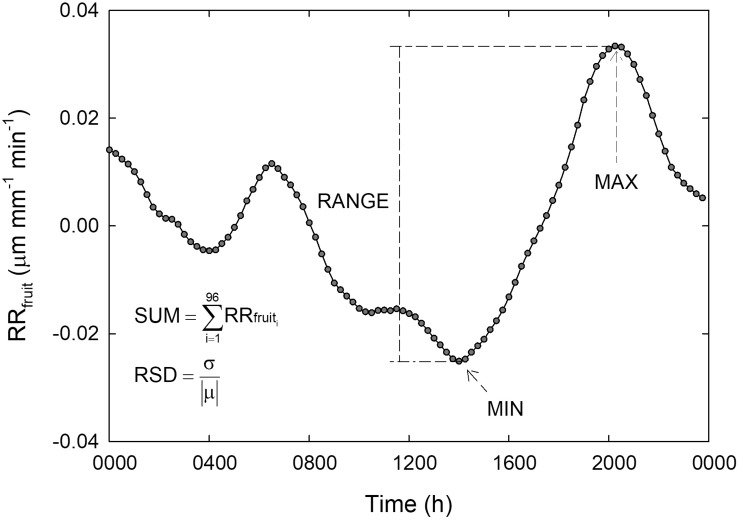
Schematization of statistical parameters calculated on a relative rate of fruit diameter change (RR_fruit_) diel curve. MIN = minimum value of RR_fruit_, MAX = maximum value of RR_fruit_, RANGE = MAX-MIN, SUM = summation of RR_fruit_ values taken at 15-minute intervals from 1 (0000 h) to 96 (2345 h), RSD = relative standard deviation, where σ is the standard deviation and —μ— is the absolute value of the mean.

Diel data were subdivided in diurnal (0600 to 2000 h) and nocturnal (2015 to 0545 h) intervals. Subsequently, diel, diurnal and nocturnal statistical parameters were extrapolated from data series for RR_fruit_ and RR_leaf_ in order to find the best predictor of midday Ψ_stem_. The parameters considered for each time interval (i.e., 24 h, night or day) were: (a) the minimum value (MIN), (b) the maximum value (MAX), (c) the summation of values at 15-minute intervals (SUM), and (d) the difference between MAX and MIN (RANGE). The relative standard deviation (standard deviation divided by the mean, RSD) was calculated as an additional parameter to allow comparison among variances expressed in different units. A graphical representation of all RR_fruit_ statistical parameters calculated on diel basis is shown in Figure 1. Similarly, all parameters were calculated for diurnal and nocturnal intervals. Raw data obtained from sensors that either caused damage to the organs or that were displaced by strong wind were discarded.

For each cultivar, the statistical parameters from nocturnal and diurnal timeframes were fitted into multiple regression models to predict midday Ψ_stem_. A backward stepwise procedure was used to discard the parameters that did not significantly contribute to Ψ_stem_ estimation.

## Results and Discussion

### Weather Conditions and Irrigation

**FIGURE 2:**
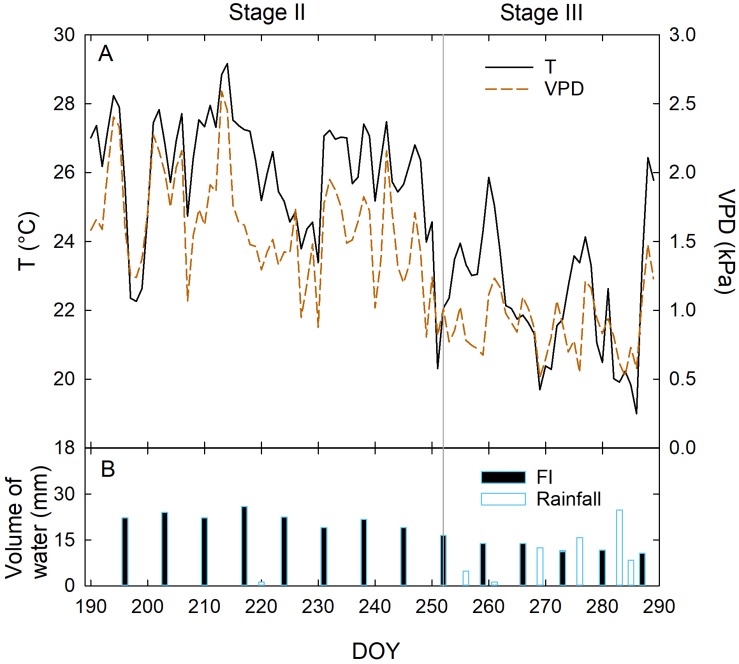
Daily mean temperature (T) and vapor pressure deficit (VPD) **(A)**, daily volumes of water supplied with full irrigation (FI) and rainfall **(B)** at fruit growth stages II and III, from 190 to 290 days of the year (DOY).

As expected, temperature (T) and vapor pressure deficit (VPD) were the highest in the measured fraction of stage II, as it occurred in full summer (Jul 8 to Sep 8) (Figure 2A). Relatively low T and VPD were recorded in stage III from 280 to 290 DOY (Figure 2A), due to frequent precipitations. In stage II, only sporadic rainfall occurred, and irrigation was approximately constant, ranging from 19 to 26 mm per week in FI trees. Lower weekly volumes of water were supplied in stage III due to the precipitations occurred from 252 to 289 DOY (Figure 2B). However, at this stage, precipitation led to an average higher weekly crop water supply (CWS, i.e., irrigation + rainfall) compared to stage II (Figure 2B). Specifically, in stage II the total CWS was mainly made up of irrigation water (Table 1), whereas precipitations at stage III contributed to the 63, 72, 83, and 100% of CWS in FI, DI-66, DI-33, and DI-0 trees, respectively.

**TABLE 1:** Crop water supply (CWS) in FI (full irrigated, 100% of crop evapotranspiration), DI-66 (66% of FI), DI-33 (33% of FI) and DI-0 (rainfed) olive trees at fruit growth stages II and III.

**Fruit growth stage**	**CWS**	**Volume of water (mm)**			
		**FI**	**DI-66**	**DI-33**	**DI-0**
II	Irrigation	197	130	64	0
	Rainfall	1	1	1	1
	Sub-total	198	131	65	1
III	Irrigation	112	74	38	0
	Rainfall	188	188	188	188
	Sub-total	300	262	226	188
Total		438	393	291	189

*Data represent averages of **‘**Nocellara del Belice’ and **‘**Olivo di Mandanici.’*

### Fruit Characteristics

**FIGURE 3:**
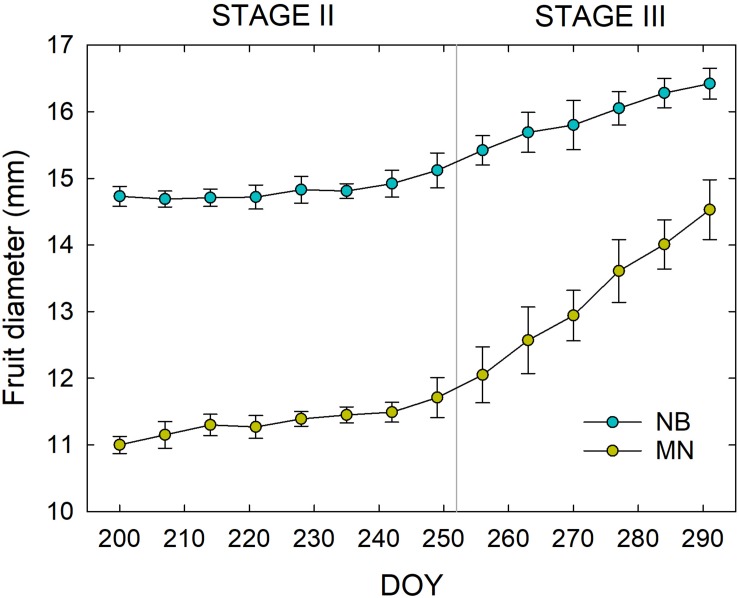
Weekly fruit diameter variations in ‘Nocellara del Belice’ (NB) and ‘Olivo di Mandanici’ (MN) olive cultivars at fruit growth stages II and III from 200 to 291 days of the year (DOY). Error bars represent standard deviations of means (*n* = 60).

The two cultivars showed different fruit shape (i.e., NB fruit were almost spherical whereas MN fruit were oblong in shape) from the beginning of fruit diameter measurements at stage II until harvest. Fruit size was also consistently greater in NB than in MN (Figure 3), with nearly no fruit growth during stage II in either cultivars, as expected in the pit hardening stage (Scalisi et al., 2019c). Stage III was characterized by a faster fruit diameter increment in MN compared to NB. The latter is related to the first part of stage III (i.e., the part shown in Figure 3), as in the remaining part of stage III until harvest, MN fruit reached an average final diameter of ∼16 mm. On the other hand, NB fruit reached an average final diameter of ∼23 mm at harvest, suggesting that they had a steeper growth after the end of the period considered in this study (i.e., after 290 DOY).

**FIGURE 4:**
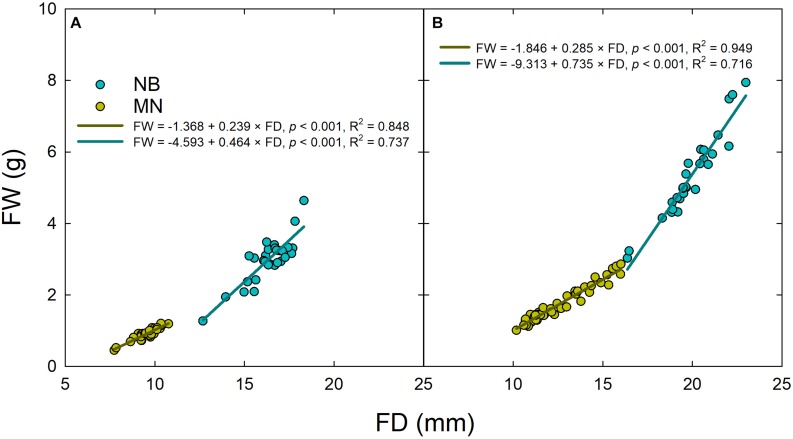
Linear regressions of fruit weight (FW) vs. fruit diameter (FD) in ‘Nocellara del Belice’ (NB) and ‘Olivo di Mandanici’ (MN) olive sampled at stage II **(A)** and stage III **(B)** of fruit development.

Linear regression analyses between fruit diameter and weight highlighted a different relationship of these two fruit parameters in both stages II (Figure 4A) and III (Figure 4B), as the slopes were significantly higher in NB than in MN (*P* < 0.001 from *t*-test) due to different pulp-stone ratios. These findings confirm once again the different fruit morphological characteristics of the two cultivars under study.

### Plant Water Status

**FIGURE 5:**
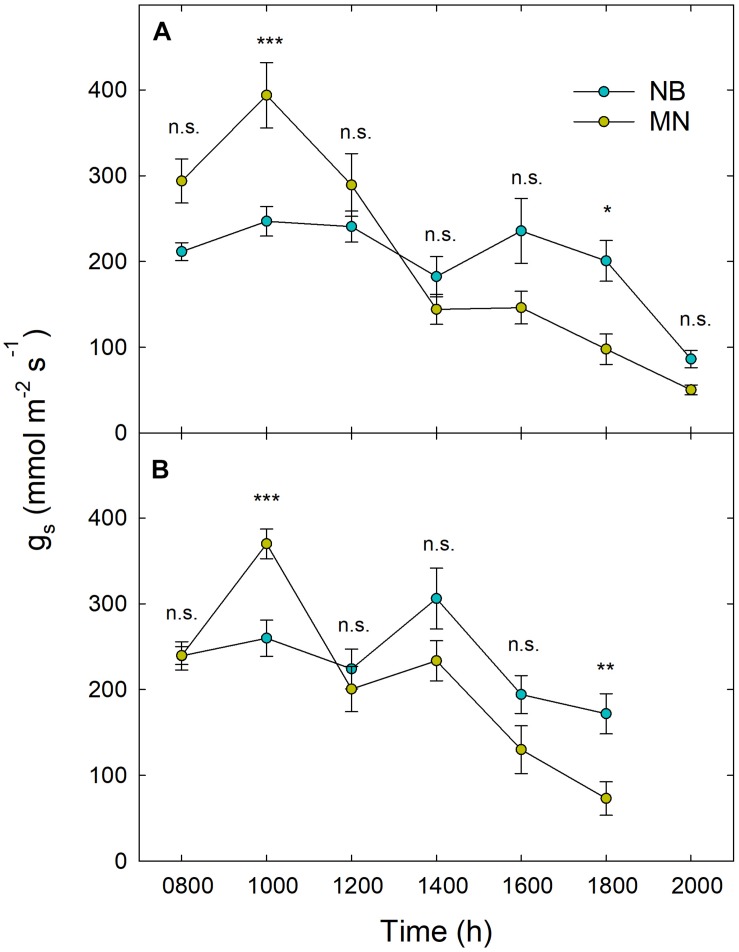
Daily curves of stomatal conductance (g_s_) in ‘Nocellara del Belice’ (NB) and ‘Olivo di Mandanici’ (MN) olive leaves at fruit growth stages II **(A)** and III **(B)**. Data averaged across different irrigation levels at 209 **(A)** and 287 **(B)** days of the year. Error bars indicate standard deviations of means. N.s., non-significant; *, significantly different for *P* < 0.05; **, significantly different for *P* < 0.01; ***, significantly different for *P* < 0.001 by Tukey’s test.

Results from daily g_s_ measurements carried out at 209 and 287 DOY did not show significantly different patterns between the 2 days of measurements and among irrigation levels. Figure 5 shows g_s_ trends in NB and MN at stage II (A) and III (B). In MN, an overall peak of g_s_ occurred at mid-morning (1000 h) with a subsequent sudden decrease at 1200 h in response to increasing noon T and VPD. On the other hand, NB leaves did not show a clear peak of g_s_ in the morning and kept stomatal aperture stable from 0800 h to 1800 h (Figures 5A,B), with a significant drop at 2000 h (Figure 5A). MN leaves showed significantly higher g_s_ than NB at 1000 h, implying a likely higher tree water consumption in the morning. This hypothesis is supported by dynamics of sap flux density measured with thermal dissipation probes in the previous year (Marino et al., unpublished), where MN trees showed higher daily water consumption, especially because of greater flows in the first part of the morning. In the afternoon, NB leaves showed higher g_s_ than MN, with significant differences occurring at 1800 h (Figures 5A,B), suggesting a tendency to maintain higher photosynthetic activity late in the day, when T and VPD are lower.

**FIGURE 6:**
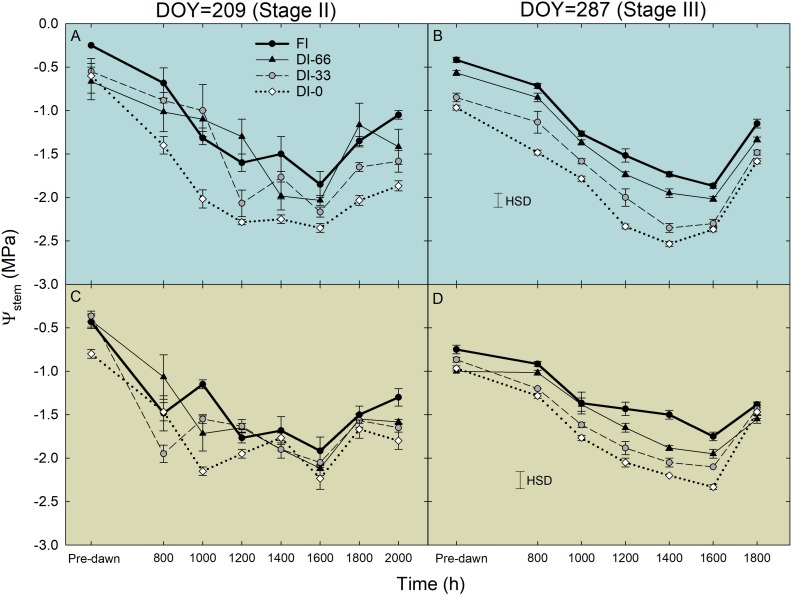
Daily curves of stem water potential (Ψ_stem_) in ‘Nocellara del Belice’ (NB) **(A,B)** and ‘Olivo di Mandanici’ (MN) **(C,D)** olive trees under full irrigation (FI), 66% of FI (DI-66), 33% of FI (DI-33) and no irrigation (DI-0) at 209 (stage II) and 287 (stage III) days of the year (DOY). Error bars represent standard deviations of means (*n* = 3). Significant differences determined by Tukey’s Honestly Significant Difference (HSD, *P* < 0.05).

Daily curves of Ψ_stem_ at stages II (209 DOY) and III (287 DOY) showed the typical overall decreasing potential at solar noon in both cultivars, with a subsequent increase late in the afternoon (Figure 6). When tested with ANOVA, only Ψ_stem_ in stage III showed significant differences among irrigation levels and a significant interaction with time (i.e., Figures 6B,D), with the lowest daily values always occurring between 1400 and 1600 h and in DI-0 trees (Figures 6B,D). During the pit hardening phase (i.e., stage II), it is well established that deficit irrigation has little effect on Ψ_stem_ (Dell’Amico et al., 2012). The minimum Ψ_stem_ was significantly lower in NB (−2.53 MPa ± 0.03) than in MN (−2.33 MPa ± 0.03, *P* < 0.001). Pre-dawn observations at stage III suggest a different behavior in the two cultivars, with only MN trees fully recovering to FI levels during the night in DI-0 and DI-33 levels (Figures 6B,D). A rise of Ψ_stem_ was observed in NB and MN trees at 1800 h, with DI-0 MN trees recovering completely to FI levels (Figure 6D).

**FIGURE 7:**
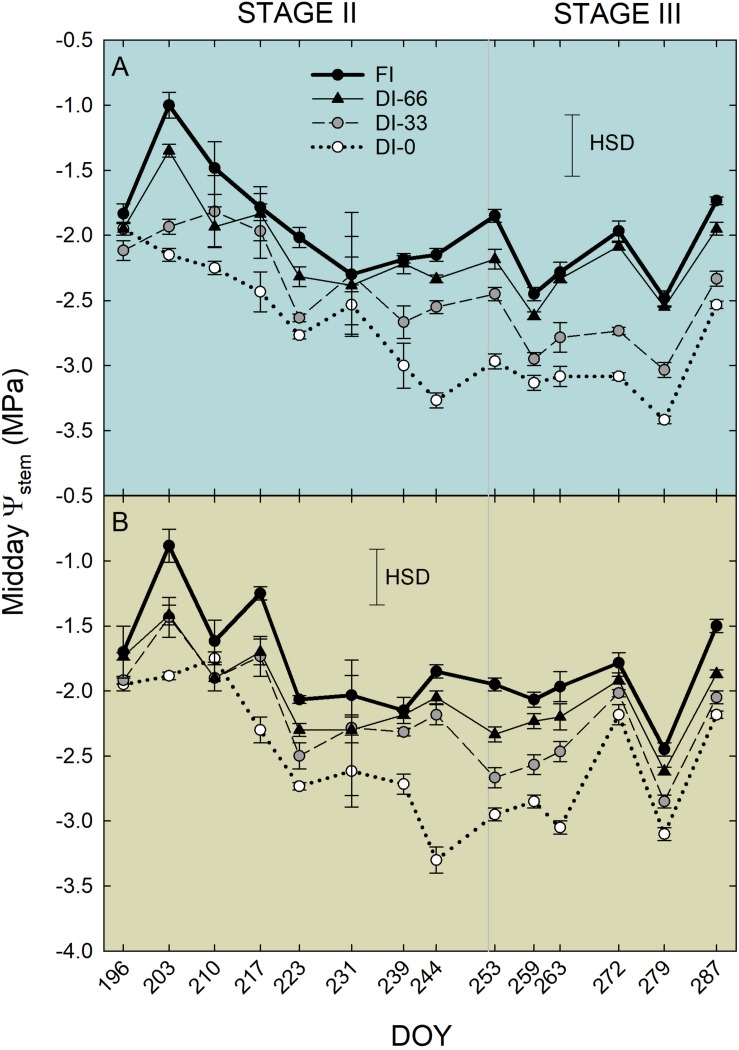
Midday stem water potential (Ψ_stem_) in ‘Nocellara del Belice’ (NB) **(A)** and ‘Olivo di Mandanici’ (MN) olive trees **(B)** under full irrigation (FI), 66% of FI (DI-66), 33% of FI (DI-33) and no irrigation (DI-0) during stage II (from 196 to 252 DOY) and stage III (from 253 to 287 DOY) of fruit growth. Error bars represent standard deviations of means (*n* = 3). Significant differences determined by Tukey’s Honestly Significant Difference (HSD, *P* < 0.05).

Weekly measurements of midday Ψ_stem_ from 196 to 287 DOY generally reflected the irrigation levels (Figure 7). Indeed, DI-0 trees experienced the lowest midday Ψ_stem_ both in stages II and III. As expected, midday Ψ_stem_ was higher at stage II than at stage III, although T and VPD were generally higher in the former (Figure 2). In this phenological phase, water deficit slows down the overall plant activity, inducing a reduction of vegetative growth and photosynthesis, which in turn might limit transpiration (Parent et al., 2009). Indeed, even g_s_ was similar in stages II and III (Figure 5), despite differences in T and VPD (Figure 2). Consequently, as water loss by transpiration is likely to be reduced, plants under deficit irrigation at stage II do not reach as low Ψ_stem_ as when the same irrigation is applied in stages I and III. The only exception occurred at 244 DOY, where DI-0 trees experienced a very low Ψ_stem_ in both cultivars, due to both particularly high T and VPD (Figure 2A) and to the transition toward the beginning of stage III which was completed the following week. In stage III, the sudden steep increases of Ψ_stem_ at 287 DOY was determined by high precipitations (Figure 2). Overall, the cultivars showed a significantly different drop of midday Ψ_stem_ in response to DI-0 (*t*-test *P* < 0.05). Indeed, across the monitoring period, rainfed NB trees showed a lower average midday Ψ_stem_ (−2.75 ± 0.07 MPa) than MN trees (−2.54 ± 0.08 MPa). Combining observations on g_s_ and Ψ_stem_, it can be said that MN avoids excessive Ψ_stem_ lowering, not by reduced stomatal aperture, as g_s_ is significantly higher in MN than in NB in the morning (Figure 5), but by other mechanisms. Despite deficit irrigation, NB trees kept their stomata open in the afternoon (Figure 5), when ET_0_ is highest, and might have not been able to limit water loss from transpiration, inducing low Ψ_stem_. Marino et al. (2016) suggested that osmotic adjustments might be responsible of the relatively higher Ψ_stem_ in a clone of MN. Furthermore, changes in the leaf cell elastic modulus occur in other olive genotypes under drought (Karamanos, 1984; Dichio et al., 2003; Bacelar et al., 2006). Both osmotic adjustments and reduced cell wall elasticity contribute to turgor preservation (Patakas and Noitsakis, 1997) and might have led to the high Ψ_stem_ found in MN.

### Fruit and Leaf-Based Sensing

**FIGURE 8:**
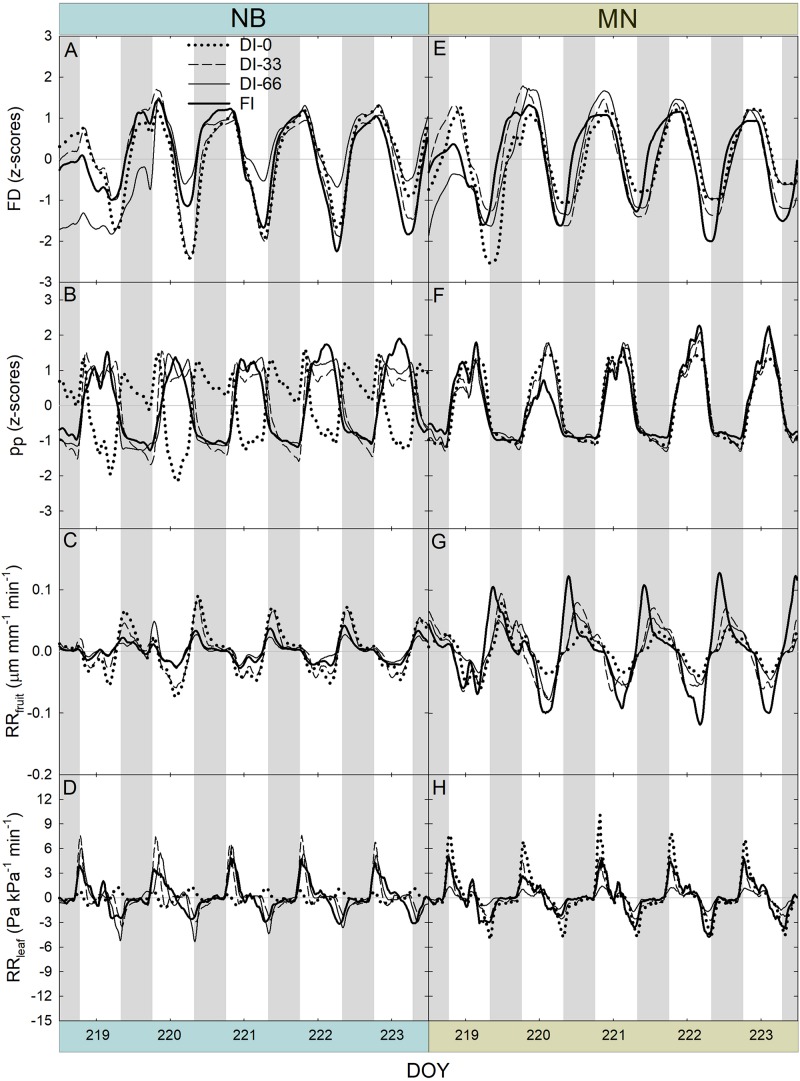
Fruit diameter (FD), leaf patch clamp pressure (p_p_), relative rate of fruit diameter change (RR_fruit_) and relative rate of leaf pressure change (RR_leaf_) recorded at 15-minute intervals for 5 days during stage II of fruit growth in ‘Nocellara del Belice’ (NB) **(A–D)** and ‘Olivo di Mandanici’ (MN) olive trees **(E–H)** under full irrigation (FI), 66% of FI (DI-66), 33% of FI (DI-33) and no irrigation (DI-0). Gray and white areas show nocturnal and diurnal hours, respectively.

After removing the three-day buffer period from FD, pp, RR_fruit_, and RR_leaf_ data, a 5-day interval in stage II was obtained (Figure 8). Trends of FD (Figures 8A,E) did not highlight different fruit growth dynamics among irrigation levels in stage II, for both NB and MN. FI induced sharper fruit shrinkage than deficit irrigation at 222 and 223 DOY, as fruit with an optimal water status are likely to exchange more water in the warmest hours of the day.

Dynamics of p_p_ highlighted the typical inversion phenomenon of the diel curve in olive leaves from trees under deficit irrigation (Fernández et al., 2011a). In NB, DI-66 and DI-33 leaves exhibited the half-inverted state (state II), whereas DI-0 leaves showed a total inversion of the curve (state III) (Figure 8B). In MN trees, a clear shift from state I to II was not observed, despite the slight tendency to enter state II at 219 and 221 DOY, with no apparent differences among irrigation levels (Figure 8F). This suggests that MN leaves can maintain high cell turgor, probably by reduced cell wall elasticity or osmotic adjustments, as found in other olive genotypes (Bacelar et al., 2009; Dichio et al., 2009; Lo Bianco and Scalisi, 2017).

The highest RR_fruit_ always occurred early in the night as fruit quickly rehydrated their tissues (Figures 8C,G). As expected, the most negative RR_fruit_ rate (i.e., the highest fruit shrinkage rate) always occurred in the warmest hours of the day. RR_fruit_ dynamics were also affected by deficit irrigation in NB, as the diel RANGE was greater in DI-0 and DI-33 than in DI-66 and FI fruit (Figure 8C). A completely different behavior was observed in MN fruit, which instead had the widest diel RANGE in FI trees (Figure 8G). In addition, the overall diel RANGE of RR_fruit_ in MN was almost double than in NB, implying larger water in- and out-flows per unit of fruit volume in the former, determined by high fruit sink power for water.

A general positive peak of RR_leaf_ was exhibited early in the morning (Figures 8D,F), representing a quick leaf turgor loss (i.e., RR_leaf_ is equivalent to the inverse of the relative change in p_c_) after pre-dawn highest turgor in the 24-hour timeframe. Even in this case, the two cultivars responded differently to water deficit, with NB DI-0 trees exhibiting minimal diel fluctuations (i.e., RANGE) while MN DI-0 trees showing the largest RANGE. This suggested that the oscillations of RR_leaf_ might be linked to those of RR_fruit_.

**FIGURE 9:**
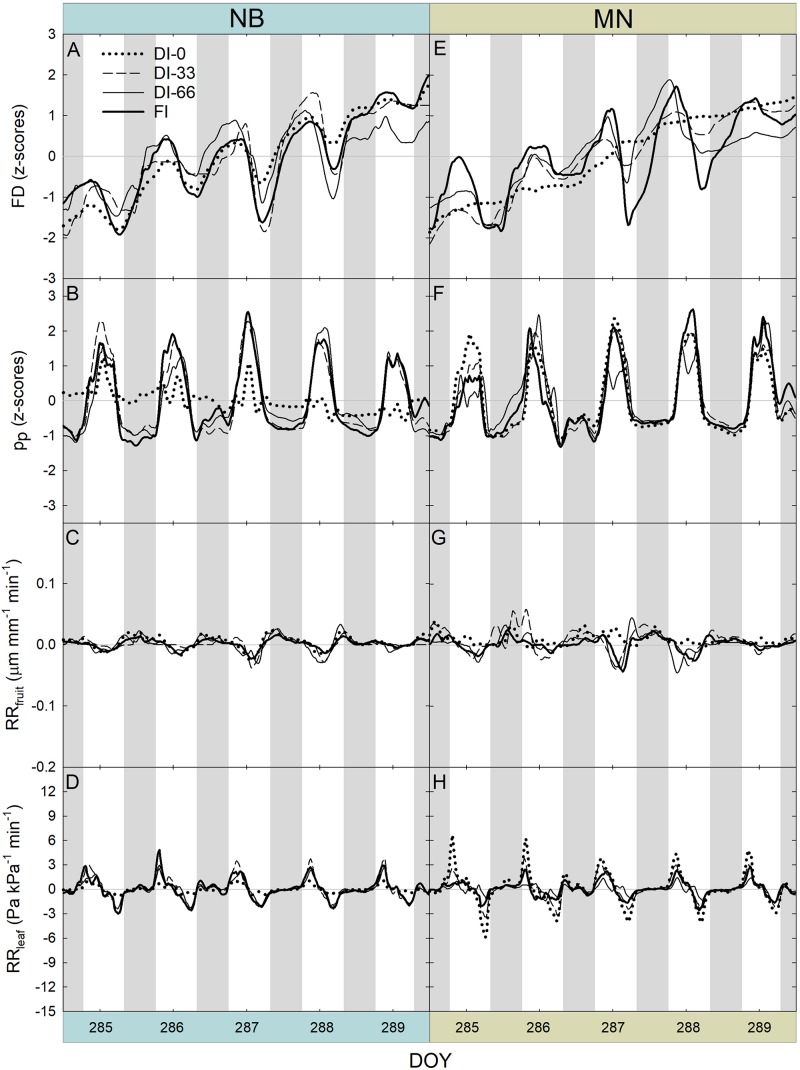
Fruit diameter (FD), leaf patch clamp pressure (p_p_), relative rate of fruit diameter change (RR_fruit_) and relative rate of leaf pressure change (RR_leaf_) recorded at 15-minute intervals for 5 days during stage III of fruit growth in ‘Nocellara del Belice’ (NB) **(A–D)** and ‘Olivo di Mandanici’ (MN) olive trees **(E–H)** under full irrigation (FI), 66% of FI (DI-66), 33% of FI (DI-33) and no irrigation (DI-0). Gray and white areas show nocturnal and diurnal hours, respectively.

Another 5-day interval was considered at stage III of fruit development (Figure 9). Differently from stage II, FD responses were characterized by an evident diameter increase across the 5 days and within the 24-hour period in both cultivars (Figures 9A,E), as in stage III fruit are in full cell enlargement phase (Figures 9A,E). Daily curves of p_p_ (Figures 9B,F) did not show pronounced inversion phenomena, as this week was characterized by high rainfall (Figure 2B) and general higher midday Ψ_stem_ (Figure 7). Only NB DI-0 trees showed a partially inverted p_p_ curve. Diel RANGE of RR_fruit_ was found to be greatly reduced at stage III (Figures 9C,G) compared to stage II (Figures 8C,G). In the former, low VPD (Figure 2A) and good soil water availability determined by abundant precipitations (Figure 2B) led to an increase of water content in fruit and lower fruit water exchanges. For similar reasons, the diel RANGE of RR_leaf_ was reduced in stage III (Figures 9D,H), although NB and MN maintained the same differences in response to deficit irrigation levels observed at stage II (Figures 8D,H).

**FIGURE 10:**
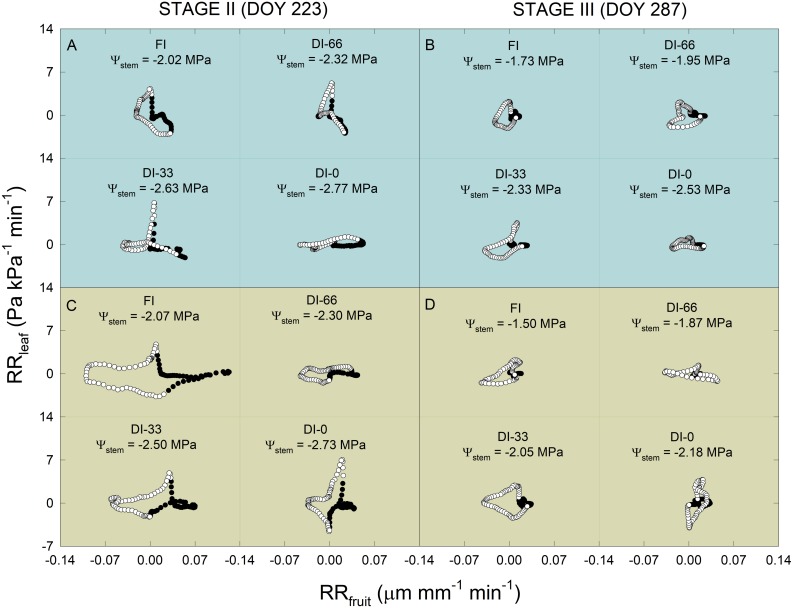
Scatter plots of relative rate of leaf pressure change (RR_leaf_) vs. relative rate of fruit diameter change (RR_fruit_) in ‘Nocellara del Belice’ **(A,B)** and ‘Olivo di Mandanici’ olive trees **(C,D)** under full irrigation (FI), 66% of FI (DI-66), 33% of FI (DI-33) and no irrigation (DI-0), at 223 (stage II) and 287 (stage III) days of the year (DOY). Midday stem water potential (Ψ_stem_) reported for each cultivar × DOY × irrigation level combination. White and black circles represent diurnal and nocturnal data in a diel interval, respectively.

Considering the interesting findings from RR_fruit_ and RR_leaf_ dynamics, these two indices were further related to each other regressing their diel data at 15-minute intervals in a day at stage II (DOY = 223) and stage III (DOY = 287). Scatter plots in Figure 10 show anti-clockwise hysteretic relationships between RR_fruit_ and RR_leaf_, both for NB (Figures 10A,B) and MN (Figures 10C,D). Hysteresis are common when relating outputs from different sensors of plant water status mounted on different organs (Tognetti et al., 1996; Fernández, 2017), as there is generally a lag in tissue water de- and re-hydration, and in our case, also a likely different pattern of the RR_leaf_ to RR_fruit_ relationship between day and night. An overall decrease of the hysteretic loop area occurred from stage II (DOY 223) to stage III (DOY 287) in both cultivars (i.e., for NB Figures 10A,B, and for MN Figures 10C,D). This is probably driven by the different fruit growth pattern at stages II and III which induced a reduction of the RR_fruit_ diel range (Figures 9C,G). In both DOY 223 and 287, the hysteretic loops in NB progressively flattened along the RR_fruit_ axis with increasing water deficit (Figures 10A,B) due to the change in the ratio between RR_fruit_ and RR_leaf_. In other words, on one hand, at increasing water deficit and in a diel interval, it seems that NB leaves significantly reduce water exchanges, as the values of RR_leaf_ stay around 0 Pa kPa^–1^ min^–1^. On the other hand, increasing water deficit caused MN loops to flatten along the RR_leaf_ axis (Figures 10C,D), with MN leaves keeping high water exchanges at low Ψ_stem_, while fruit water exchanges were significantly reduced, as RR_fruit_ did not change much from 0 μm mm^–1^ min^–1^. This opposite trend suggests a completely different mechanism of leaf and fruit water exchanges in response to increasing water deficit in the two cultivars, which might be driven by different osmotic adjustments, cell-wall elasticity and tissue water content.

The statistical diel, nocturnal and diurnal parameters of RR_fruit_ (i.e., MIN, MAX, SUM, RANGE, and RSD) were associated to the corresponding RR_leaf_ parameters to assess fruit and leaf responses to water deficit. Subsequently, data were analyzed by MANOVA to determine whether the combined response of parameters was affected by cultivars, irrigation levels, and cultivar × irrigation interaction. The cultivar did not influence significantly diel, diurnal and nocturnal RR_fruit_/RR_leaf_ when statistical parameters were considered together (Table 2). Diel and diurnal RR_fruit_/RR_leaf_ parameters changed significantly in response to irrigation levels, but the cultivar × irrigation interaction had the strongest effect (Table 2), indicating that RR_fruit_/RR_leaf_ responses to deficit irrigation differ between the two genotypes under study. Specifically, the highest F was found in the MANOVA that tested diurnal RR_fruit_/RR_leaf_ responses to cultivar × irrigation. These results suggest that, under increasing water deficit, the differences in genotype-specific fruit and leaf sink power to water are predominant in day hours.

**TABLE 2:** Multivariate analyses of variance (MANOVAs) testing the effects of cultivar, irrigation and the cultivar × irrigation interaction on the ratios of statistical parameters extrapolated from the relative rate of fruit diameter change (RR_fruit_) and the relative rate of leaf pressure change (RR_leaf_) at diel, diurnal and nocturnal intervals.

**Factor**	**Time frame**	**Significance level for Wilk’s Lambda test**	**F**
Cultivar	Diel	0.495	0.894
	Diurnal	0.260	1.365
	Nocturnal	0.104	1.991
Irrigation	Diel	0.025	1.965
	Diurnal	0.001	2.783
	Nocturnal	0.238	1.266
Cultivar × Irrigation	Diel	<0.001	5.195
	Diurnal	<0.001	6.515
	Nocturnal	0.031	1.907

*Each line represents a single MANOVA based on the following ratios of statistical parameters: MIN = MIN(RR_fruit_)/MIN(RR_leaf_); MAX = MAX(RR_fruit_)/MAX(RR_leaf_); SUM = SUM(RR_fruit_)/SUM(RR_leaf_); RANGE = [(MAX(R_fruit_) – MIN (RR_fruit_)]/[(MAX(RR_leaf_) – MIN(RR_leaf_)]; and RSD = RSD(RR_fruit_)/RSD(RR_leaf_). Significance levels and F shown for each MANOVA.*

**FIGURE 11:**
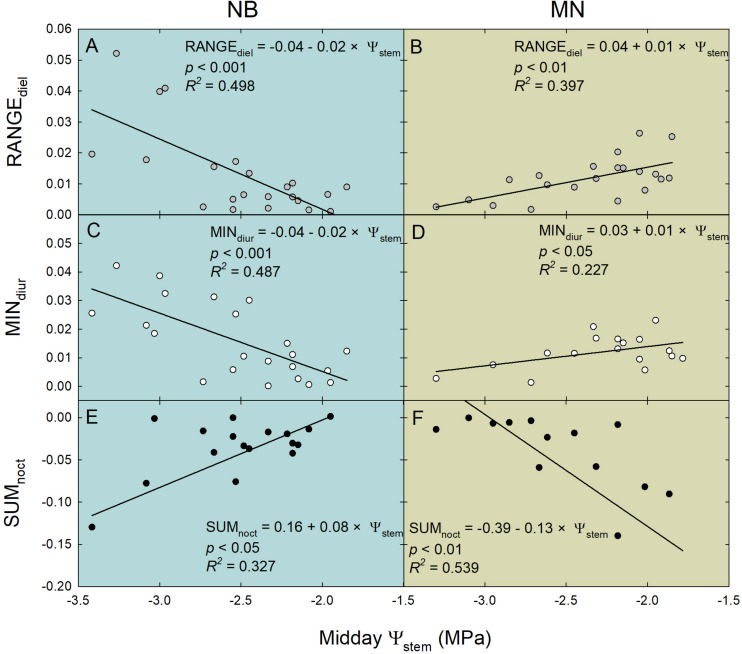
Linear relationships of ratios between the statistical parameters of the relative rate of fruit diameter change (RR_fruit_) and the relative rate of leaf pressure change (RR_leaf_) vs. midday stem water potential (Ψ_stem_). RANGE_diel_ = diel [RANGE(RR_fruit_)/RANGE(RR_leaf_)] **(A,B)**; MIN_diur_ = diurnal [MIN(RR_fruit_)/MIN(RR_leaf_)] **(C,D)**; SUM_noct_ = nocturnal [SUM(RR_fruit_)/SUM(RR_leaf_)] **(E,F)**. Gray, white and black circles represent diel, diurnal and nocturnal data, respectively, for “Nocellara del Belice” (NB) and “Olivo di Mandanici” (MN) olive trees.

Ratios between RR_fruit_ and RR_leaf_ diel, diurnal and nocturnal parameters were often linearly related to midday Ψ_stem_ (Figure 11). Interestingly, linear regression models highlighted opposite trends in the two cultivars for many of the several associations tested. In NB, RANGE_diel_ [diel RANGE(RR_fruit_)/diel RANGE(RR_leaf_)] was higher at lower midday Ψ_stem_, suggesting more marked water exchanges (e.g., in and out) in fruit rather than leaves at pronounced water deficit (Figure 11A). An opposite trend was observed in MN, in which decreasing midday Ψ_stem_ lead to higher leaf water exchanges (Figure 11B). This inverted trend agrees with RR_fruit_ and RR_leaf_ fluctuations shown in Figures 8C,D,G,H. In NB, during the day MIN_diur_ [diurnal MIN(RR_fruit_)/diurnal MIN(RR_leaf_)] increased along increasing water deficit (Figure 11C). This indicates that as water deficit increases, the highest diurnal speed of fruit water loss becomes higher compared to the highest speed of leaf turgor gain. Even in this case, an opposite trend is found in MN, with the diurnal rate of fruit water loss being higher than leaf rehydration rate at low midday Ψ_stem_ (Figure 11D). In NB, SUM_noct_ [nocturnal SUM(RR_fruit_)/nocturnal SUM(RR_leaf_)] decreased with increasing water deficit, with leaf rehydration being favored over fruit water gain (Figure 11E). Oppositely, in MN the SUM_noct_ ratio was inversely related to midday Ψ_stem_ (Figure 11F), suggesting a stronger nocturnal sink power for water of fruit compared to leaves under more severe water deficit.

All the insights obtained so far suggest that fruit to leaf relationships in terms of RR_fruit_ and RR_leaf_ dynamics at different times of the day can be strictly related to midday Ψ_stem_, thus, to plant water status.

The backward stepwise multiple regression analysis for NB indicated that most of the statistical parameters significantly contributed to the model in Eq. 3 (*P* < 0.05), and only MIN_diur_, RSD_diur_ [diurnal RSD(RR_fruit_)/diurnal RSD(RR_leaf_)] and MIN_noct_ [nocturnal MIN(RR_fruit_)/nocturnal MIN(RR_leaf_)] were discarded. RSD_noct_ [nocturnal RSD(RR_fruit_)/nocturnal RSD(RR_leaf_)] provided the greatest contribution to the model (*P* < 0.001, *F* = 29.400). In the multiple regression model obtained for MN (Eq. 4), only four parameters were significant (*P* < 0.05): RANGE_diur_ [diurnal RANGE(RR_fruit_)/diurnal RANGE(RR_leaf_)], RSD_diur_, MIN_diur_ and RSD_noct_. Also in this case, RSD_noct_ represented the parameter exhibiting the most relevant effect to the prediction of midday Ψ_stem_ (*P* < 0.001, *F* = 24.569).

(3) NBΨ=stem-1.96+(65.54×MAX)diurr

-(106.84×RANGE)diurs-(21.27×SUM)diurt

-(3.20×MAX)noctu+(89.56×RANGE)noctv

+(25.96×SUM)noctw-(0.365×RSD)noctx

(P<0.001,R=20.924,S.E.=0.14)

(4) MNΨ=stem-1.35-(40.01×RANGE)diur

-(0.11×RSD)diury-(21.649×MIN)diurz

-(0.280×RSD)noct(P<0.001,R=20.879,

S.E.=0.17)

where, ^*r*^ = diurnal [MAX (RR_fruit_)/MAX (RR_leaf_)]; ^*s*^ = diurnal [RANGE (RR_fruit_)/RANGE (RR_leaf_)]; ^*t*^ = diurnal [SUM (RR_fruit_)/SUM (RR_leaf_)]; ^*u*^ = nocturnal [MAX(RR_fruit_)/MAX (RR_leaf_)]; ^*v*^ = nocturnal [RANGE (RR_fruit_)/RANGE (RR_leaf_)]; ^*w*^ = nocturnal [SUM (RR_fruit_)/SUM (RR_leaf_)]; ^*x*^ = nocturnal [RSD (RR_fruit_)/RSD (RR_leaf_)]; ^*y*^ = diurnal [RSD (RR_fruit_)/RSD (RR_leaf_)]; ^*z*^ = diurnal [MIN (RR_fruit_)/MIN (RR_leaf_)].

A similar highly significant relationship of RSD_noct_ and midday Ψ_stem_ was also observed in nectarine (Scalisi et al., 2019c), and in both cases (i.e., olive and nectarine), the relationship of RSD_noct_ to Ψ_stem_ was negative (RSD_noct_ linear components in Eqs. 3, 4), suggesting that the variability of RR_fruit_ at lower Ψ_stem_ is higher than the one of RR_leaf_. In other words, being the night the fraction of the 24 h at which both fruit and leaf tissues import water, when trees are severely stressed, fruit water import are relatively higher than leaf’s when compared to the same relationship in no water-deficit conditions. However, the two olive cultivars under study show different patterns of fruit and leaf water-exchange regulation at other times of the day under increasing water deficit gradients. NB seems to favor fruit water exchanges over leaf’s when water deficit increases (Figure 11A), as indicated by the wide fluctuations of fruit water in- and out-flows in trees under DI-0 conditions (Figure 8C). At the same time, DI-0 leaves reduce their transpiration and water in-flow leading to minimum turgor gain (Figure 8D). On the contrary, MN leaf water exchanges become predominant compared to fruit under water deficit conditions, whereas fluctuations of fruit water in- and out-flows are wider than leaf’s in FI trees (Figures 8G,H, 11B). This differentiation in the response to drought is likely to be due to both fruit and leaf characteristics. Indeed, the water potential in NB leaves is likely to go negative, as suggested by daily g_s_ (Figure 5) and Ψ_stem_ (Figures 6A,B) trends. This leads to a higher loss of leaf cell turgor in NB, despite similar midday Ψ_stem_ to MN (Figure 7), as confirmed by the inversion of the p_p_ curve at 223 DOY (Figures 8C,D). In turn, the low turgor leads to a decrease of RR_leaf_ diel fluctuations. The most pronounced loss of turgor in NB leaves might also be driven by a lower leaf cell-wall elasticity in this cultivar than in MN, a trait that Bacelar et al. (2009) associated to less drought-tolerant genotypes. Concomitantly, NB fruit increase their RR_fruit_ fluctuations in DI-0 conditions, perhaps driven by high cell-wall elasticity, acting as the main pump of water, fruit-to-leaf vascular flows. The generally significantly higher midday Ψ_stem_ exhibited by MN can also be associated to a relatively higher water potential in the leaves compared to NB, as also in this case stomatal regulation did not differ among irrigation levels. The hypothesized higher leaf osmotic adjustments and cell-wall elasticity in MN under drought may also be confirmed by the low tendency to an inversion of the p_p_ curve (Figure 8F). In addition, the positive relationship between RANGE_diel_ and midday Ψ_stem_ in MN (Figure 8B) is determined by the lower fruit water exchanges at DI-0 levels (Figure 8G), which may be driven by lower fruit cell-wall elasticity in MN compared to NB.

The different mechanisms in the two cultivars were observed in an -ON year, and it would be interesting to further study tree responses in -OFF years, as Dell’Amico et al. (2012) and Girón et al. (2015) observed that in “Manzanillo” trees crop load influenced the ability of fruit and leaves to act as sinks for water. Changes in osmotic adjustments (Dichio et al., 1997, 2009; Lo Bianco and Scalisi, 2017) and cell-wall elasticity (Xiloyannis et al., 1993; Bacelar et al., 2009) along water deficit gradients have been reported for leaves. However, to the best of our knowledge, no consideration has been previously given to concomitant changes of similar drought tolerance mechanisms in fruit. Divergent physiological responses of fruit and leaf to drought might also be affected by different productive performance, fruit number and tree vigor. At harvest, MN showed significantly higher yield (Y = 7.30 ± 0.60 kg/tree), trunk cross-sectional area (TCSA = 51.3 ± 1.97 cm^2^), yield efficiency (YE = 0.14 ± 0.01 kg cm^–2^) and crop load (CL = 4562 ± 615 n. of fruit) compared to NB (Y = 3.78 ± 0.66 kg/tree, TCSA = 41.37 ± 1.49 cm^2^, YE = 0.09 ± 0.01 kg cm^–2^ and CL = 510 ± 100 n. of fruit). The greatly higher number of fruits in MN may represent a conspicuous water reservoir for the tree and be involved in a buffer effect on plant response to drought. NB compensates its low fruit number with an average fruit water content six-fold higher than MN (data not shown), for a total tree water storage in fruit tissues almost equivalent to MN trees. A divergent evolution of the two genotypes may have favored the development of diversified protective mechanisms that control fruit and leaf water exchanges under drought.

## Conclusion

The results of this work suggest that overall a lower amount of water can be used for irrigation in MN, as this cultivar tends to lose leaf cell turgor at lower Ψ_stem_ than NB and therefore can be considered a drought-tolerant genotype. The behavior of NB is instead more compatible with drought-avoidance, as leaf stomatal conductance was moderate compared to MN, and in accordance with Scalisi et al. (2019a) who reported an increased root/leaf biomass ratio in NB under drought. Our findings reveal different strategies of fruit and leaf water exchanges in droughted trees, suggesting that olive genotypes can favor the water status of one organ over the other in conditions of water scarcity. The use of genotype-dependent models is therefore essential to determine how leaf and fruit water exchanges can be related to plant water status. These models can provide the basis for an automated modulation of irrigation in response to pre-defined thresholds of water deficit. The two models described in Eqs. 3, 4 can be used for a precise day-by-day assessment of midday Ψ_stem_ in the two cultivars under study, and in -ON years. Future studies should integrate responses from stage I in the multiple regression models.

Overall, this work confirms the advantages of combining fruit and leaf water dynamics for the prediction of plant water status in olive, whose suitability was just confirmed in a nectarine study (Scalisi et al., 2019c). Nevertheless, technologies that sense fruit and leaves water dynamics are still independent and need to be fit in a unique system for real-time monitoring. The use of this combined approach in other fruit species of horticultural interest is highly recommended, despite the complexity of a correct management of a large network of sensors.

## Data availability statement

The raw data supporting the conclusions of this article will be made available by the authors, without undue reservation, to any qualified researcher.

## Author Contributions

All authors contributed to conception and design of the study. FM, TC, and RL made available the equipment used in the experiment. AS carried out the field measurements and wrote the first draft of the manuscript. AS and RL performed the statistical analysis. GM, FM, TC, and RL contributed to the data interpretation. All authors contributed to manuscript revision, read and approved the submitted version.

## Conflict of Interest

The authors declare that the research was conducted in the absence of any commercial or financial relationships that could be construed as a potential conflict of interest.
